# Treatment of canine oral papillary squamous cell carcinoma using definitive‐intent radiation as a monotherapy—a case series

**DOI:** 10.1111/vco.12646

**Published:** 2020-10-01

**Authors:** Francine van der Steen, Maurice Zandvliet

**Affiliations:** ^1^ Utrecht University Animal Cancer Center (UUACC), Department Clinical Sciences, Faculty of Veterinary Medicine Utrecht University Utrecht Netherlands

**Keywords:** canine, oral, papillary squamous cell carcinoma, radiation therapy

## Abstract

Canine oral papillary squamous cell carcinoma (COPSCC) is a rare neoplasm and although locally invasive it carries a favourable prognosis following wide surgical excision. Radiotherapy has been reported to be effective as an adjunct treatment to surgery. However, limited information is available on the role of radiotherapy as single treatment. This single‐institution retrospective study describes a series of 10 dogs diagnosed with macroscopic COPSCC that were treated with definitive‐intent radiotherapy (DRT) as a monotherapy. These dogs had a median age of 4 years (range: 0.4‐9.6 years). The tumour was located in the rostral oral cavity in all cases with a median tumour size of 2.5 cm (range: 0.8‐6.8 cm). No local or distant metastases were identified. All dogs were treated with electron beam DRT (>32Gy, 10‐16 daily fractions of 3.2Gy). The median follow‐up time was 961 days (range: 333‐3.498 days) with nine dogs achieving a complete response and one dog a partial response. The dog with the partial response developed disease progression at 228 days after initiation of radiotherapy. Two dogs died from non‐tumour‐related causes. The remaining seven dogs were still alive and in complete remission at the time of last follow‐up. Median progression‐free survival time and median survival time were not reached. DRT was generally well tolerated, but all dogs experienced self‐limiting acute radiation mucositis (grade 2‐3) and/or dermatitis (grade 1). No late radiation toxicity was observed. Macroscopic COPSCC appears to be a radiosensitive tumour that can be successfully treated with DRT eliminating the need for aggressive surgery in advanced cases.

**Abbreviations**
COPSCC: canine oral papillary squamous cell carcinoma
DRT: definitive‐intent radiotherapy
Gy: grey
SCC: squamous cell carcinoma

## 1 INTRODUCTION

Oral cancer is amongst the most common types of canine neoplasia and affects dogs of all ages and breeds.^1^ Various types of neoplasia have been reported, but squamous cell carcinoma is the second most common histologic diagnosis.^1^, ^2^ Canine oral papillary squamous cell carcinoma (COPSCC) represents a distinct histological subtype of canine oral squamous cell carcinoma (SCC).^3^ COPSCC is a rare and locally invasive neoplasm that can affect very young dogs but has also been reported in adult dogs.^3^, ^4^, ^5^ It has a predominantly exophytic papillary growth pattern,^2^ and contrary to what its name might suggest, there is no documented relation with a papillomavirus infection.^6^, ^7^ Physical examination, diagnostic imaging and routine histological examination do not always allow for a clear differentiation between COPSCC and conventional SCC or even acanthomatous ameloblastoma.^3^, ^5^ Additional immunohistochemistry may be required but is still no guarantee for an unequivocal diagnosis.^7^

COPSCC has a low metastatic potential.^4^ Therefore, COPSCC usually has a favourable prognosis following wide surgical excision.^5^, ^6^, ^8^ However, alternative treatment options are needed for dogs with large invasive COPSCC that require aggressive surgical treatment. Aggressive oral surgery can be technically challenging, may affect the dog's oral function or its appearance, or just simply be declined by its owner. Radiotherapy has been used in a small case series as an adjunct treatment to incomplete resected COPSCC and resulted in durable local tumour control.^6^ However, there are limited data available on the use of radiation therapy in the treatment of COPSCC and to the authors' knowledge, there are no published studies evaluating the use of radiation therapy in COPSCC as a primary treatment.

The objective of this study was to describe the outcome and toxicity of a regularly fractionated definitive‐intent radiation therapy protocol as a monotherapy for dogs with macroscopic COPSCC.

## 2 MATERIAL AND METHODS

### 2.1 Patient selection

Records of dogs with a histopathological diagnosis of oral PSCC that were referred to Utrecht University Animal Cancer Center and had received definitive‐intent radiation therapy between May 2010 and December 2019 were retrospectively reviewed. Dogs that received prior treatment with surgery and had no macroscopic disease at presentation were excluded from this study. For each dog, the following data were recorded: signalment, tumour location, tumour size, staging results (imaging and cytology), radiation therapy protocol, treatment response, treatment‐related toxicity and outcome. Radiographs and computed tomography (CT) images were reviewed when available. The World Health Organization (WHO) staging system for canine oral tumours^9^ was used to determine stage.

### 2.2 Radiation therapy

The reporting guidelines for veterinary radiation oncology of Keyerleber et al. (2012)^10^ were used for description of the radiation therapy. All dogs were treated with external beam megavoltage radiation therapy using a linear accelerator (Elekta Precise Treatment System, Crawley, UK). The intended total dose was 48 Gy administered over 15 daily fractions by a single ipsilateral portal using electron beam radiation.

To ensure consistent patient positioning vacuum cushions (SecureVac, Bionix, Toledo) and individualized mouth gags were used. Patient position was dependent on tumour location with patients being treated in lateral (tumours in lateral aspect maxilla or mandible), dorsal (rostral maxillary tumours) or ventral (rostral mandibular tumours) recumbency. The central axis of the electron beam was perpendicular to the tumour and the gantry was angled such that a maximum contact area between electron beam applicator and tumour surface was obtained. The resulting gantry and collimator angles were used for each following treatment. The electron beam applicator was in direct contact with the tumour surface resulting in a source to surface distance (SSD) of 100 cm.

In all cases, manual planning was performed based on gross tumour volume (GTV) as assessed by clinical examination and diagnostic imaging findings. For the extension to clinical target volume (CTV) and planning target volume (PTV) a combined minimal margin of 1 cm in all directions was used. Margin orientation was related to dental elements and no port films were taken. The size of the electron beam applicator was determined by the PTV and in most cases, a 6 × 6 cm^2^ field beam applicator was used. Depending on the individual tumour size, Cerrobend inlays were used to reduce the effective field size. No clinically relevant organs at risk were included within the radiation field.

Electron beam strength used was based on tumour depth (deepest tumour dimension along the central beam axis + 1 cm margin) and was selected based on the 95% depth dose, which was 3.0 and 3.5 cm for 12 and 15 MeV, respectively. Commercial tissue equivalent material (Elasto‐Gel, Southwest technologies, North Kansas City) and wet gauze were used to allow for adequate dose build‐up and in smaller tumours tissue equivalent material was used to limit the beam's depth of penetration.

The dose (monitor‐units) was calculated through multiplying the dose (cGy) by the machine‐specific Field(−size)‐factor.

Dosimetry checks were performed on a weekly basis using the PTW Quickcheck (Quickcheck, PTW, Freiburg, Germany) and a complete physics check using a water phantom was performed twice‐yearly by certified clinical physicist. In case of abnormalities detected on the weekly checks additional physics checks were performed. No in vivo dosimetry was performed.

Dogs were anaesthetised for every treatment. Anaesthetic protocols consisted, in most cases, of an opioid with (dex)medetomidine premedication, followed by propofol induction and maintenance with propofol or isoflurane gas.

Three weeks following completion of the radiation treatment all dogs were rechecked by the radiation oncologist and in between supportive treatment was provided based on the information obtained from the owner.

### 2.3 Treatment efficacy

Tumour response was determined by measurement of the macroscopic tumour and classified according to the Veterinary Cooperative Oncology Group RECIST guidelines for dogs.^11^ A complete response was defined as complete disappearance of the target lesion. A partial response was defined as a minimum of 30% reduction in the longest diameter of the target lesion compared with baseline. Stable disease was defined as less than 30% reduction or 20% increase in the longest diameter of the target lesion compared with baseline. Progressive disease was defined as at least a 20% increase in the diameter of the target lesion compared with baseline. In all dogs, the treatment response was evaluated during the radiotherapy treatment on a daily basis and at recheck 3 weeks later. Information on long‐term outcome was obtained from the medical records and by telephone interview with the owner or referring veterinarian.

### 2.4 Toxicity assessment

Radiation‐induced toxicity was assessed through review of the patient findings as recorded by the treating veterinary oncologist listed in the medical file and by telephone call with the owners and referring veterinarians. Acute and late effects were characterized according to the radiation morbidity scoring described by the Veterinarian Radiation Therapy Oncology Group.^12^ Radiation‐induced toxicity that developed during the first 6 months after initiation of the radiotherapy was classified as acute toxicity, whilst those that developed 6 months or more after the initiation of radiation therapy were classified as late toxicity.

### 2.5 Statistical analysis

Progression free survival and overall survival were calculated using the Kaplan‐Meier product‐limit method. Progression free survival was defined as the time in days from the date of the initiation of radiation therapy until tumour recurrence or progressive disease. Overall survival was defined as the time in days from the date of the initiation of radiation therapy until death. Animals that were progression‐free and alive at the time of data analysis or had died from non‐tumour or non‐therapy related causes were censored. None of the dogs was lost to follow‐up. Statistical analyses were performed using the IBM SPSS Statistics V22.0 (IBM Corp, Armonk New York) software package.

## 3 RESULTS

### 3.1 Patient data

Ten dogs met the inclusion criteria for the study. Results for signalment, tumour size and location are summarized in Table 1. The median age at the start of radiation treatment was 4 years (range: 0.4‐9.6 years) and four out of 10 dogs were less than 1 year of age. The median body weight was 24.6 kg (range: 6.7‐66.8 kg) and included a total of three female and seven male dogs. Two dogs were mixed‐breed whilst the remaining eight dogs were pure‐bred (Table 1).

**TABLE 1:** Clinical features of the 10 dogs with oral papillary squamous cell carcinoma

Case no.	Breed	Sex	Age (Y)	Weight (kg)	Maximum tumour size (cm)	Tumour location
1	American bulldog	M	0.4	26.2	2.5	Rostral maxilla
2	American Staffordshire terrier	M	0.6	23	3.3	Right rostral maxilla
3	South African mastiff	MN	3.0	59.4	2.5	Right rostral maxilla
4	Friesian stabyhoun	FN	6.0	17	1.5	Left rostral maxilla
5	Mixed‐breed	F	9.0	8.7	2.1	Rostral maxilla
6	Leonberger	MN	5.0	66.8	0.8	Left rostral mandible
7	Nova scotia duck tolling retriever	FN	7.5	17.6	2.8	Right rostral mandible
8	Labradoodle	MN	9.6	36.3	1.6	Right rostral mandible
9	Mixed‐breed	MN	0.7	6.7	2.5	Right rostral maxilla
10	Bernese mountain dog	M	0.6	30.1	6.8	Right rostral mandible

Abbreviations: F, female; FN, female neutered; M, male; MN, male neutered.

The median tumour size (longest diameter) was 2.5 cm (range: 0.8‐6.8 cm). The tumour affected the rostral oral cavity in all dogs: two were located in the rostral maxilla, three in the right maxilla, one in the left maxilla, one in the left mandible, and three in the right mandible.

In four dogs, complete blood count and serum biochemical analysis were performed at the time of initial examination and revealed no abnormalities.

In all dogs, the diagnosis of COPSCC was confirmed by means of a histological biopsy and all biopsies were examined by board‐certified veterinary pathologists from different commercial pathology laboratories.

### 3.2 Imaging characteristics of the primary tumour

Computed tomography of the head was performed in all cases except one (Case 7).

**FIGURE 1:**
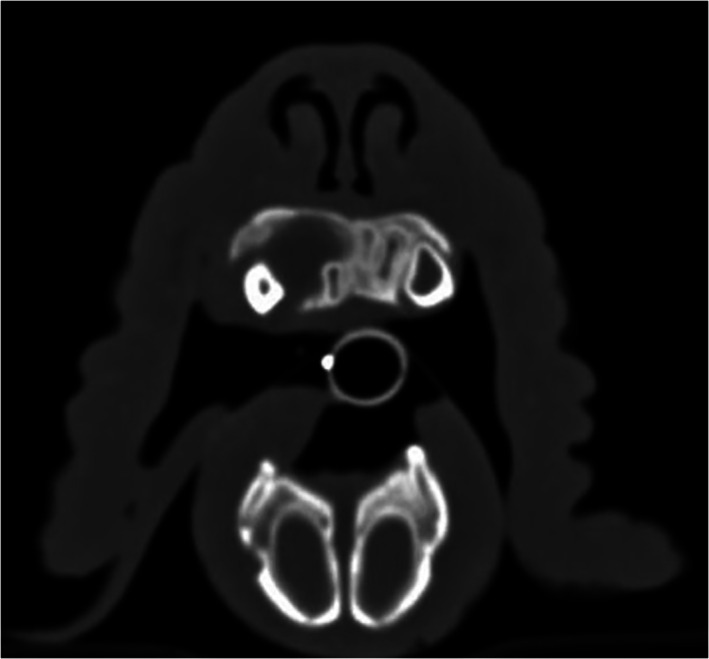
Computer tomographic image from Case 1. Note the expansile cyst‐like lesion at the right incisive bone. This cyst‐like lesion is consistent with a cavitating pattern. (Image courtesy of the Division of Diagnostic Imaging, Veterinary Faculty, Utrecht University)

**FIGURE 2:**
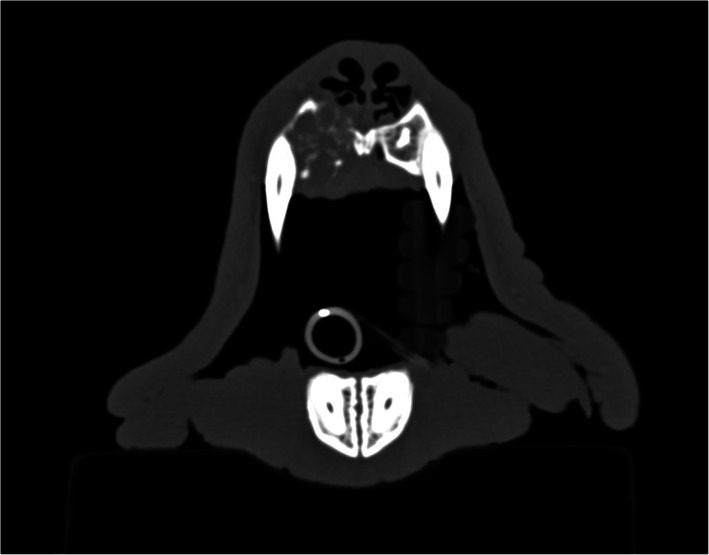
Computer tomographic image from Case 3. Note the infiltrative mass at the level of the right canine tooth with destruction of the incisive bone and invasion of the mass into the nasal cavity. This infiltrative lesion is consistent with a non‐cavitating pattern. (Image courtesy of the Division of Diagnostic Imaging, Veterinary Faculty, Utrecht University)

In Case 7, only x‐rays of the skull were available for reassessment. Of the nine dogs that underwent contrast‐enhanced CT, the most common CT features were the presence of a soft‐tissue mass lesion (9/9), osteolysis (9/9), contrast enhancement of the mass lesion (5/9) and loco‐regional lymphoadenomegaly (4/9). As described by Nemec et al. (2014),^4^ five cases in this study on CT imaging have a cyst‐like pattern of bone loss or an expansile mass consistent with a cavitating form (Figure 1) and four cases an infiltrative pattern of bone destruction consistent with a non‐cavitating form (Figure 2).

### 3.3 Staging

In three cases, the tumour measured <2 cm (T1), in six cases 2 to 4 cm (T2) and in one case the tumour was >4 cm in maximum diameter (T3). On computed tomography of the regional lymph nodes (9/10) and thorax (5/10) there is no evidence of pulmonary metastasis in any of the cases. Aspiration biopsies on four cases with enlarged lymph nodes reported a diagnosis of reactive lymphoid hyperplasia on cytological evaluation. According to the WHO classification system for oral tumours three dogs were classified as stage I, six dogs as stage II and one dog as stage III.

### 3.4 Treatment

In all dogs, macroscopic disease was present at the start of radiation therapy. The radiation protocol was administered as planned in all but two dogs (Case 1 and Case 10) that received a shorter course. Case 1 received 14 fractions (44.8 total dose) and Case 10, 10 fractions (32 Gy total dose) and this was in both cases because of rapid and near‐complete response of the tumour within the first week of radiation therapy. In Case 10, the radiation protocol was significantly shorter (10 fractions) than intended because of the combination of obtaining a complete response following five radiation fractions, the need for additional surgery (in an attempt to reduce the risk for radiation‐related complications with wound healing), and financial constraints for the owner. In Case 3, there was an unintended treatment break because of technical issues with the linear accelerator that was compensated for with an additional 16th fraction leading to a total dose of 51.2 Gy. The treatment protocol for each individual dog is shown in Table 2. None of the dogs received any form of medication at start of radiation therapy.

**TABLE 2:** Treatment and outcome of a regularly fractionated definitive‐intent radiation therapy protocol in the 10 dogs with oral papillary squamous cell carcinoma

Case no.	Total dose (Gy)	Fractions	Treatment response	PFS (days)	Overall survival (days)	Status at last follow‐up	Toxicity–acute
1	44.8	14	CR	3.498	3.498	Alive	Mucosa (grade 3)
2	48	15	CR	1.826	1.826	Euthanasia for behavioural disorder‐NTR	Mucosa (grade 2)
3	51.2	16	CR	730	730	Euthanasia for neurological disorder‐NTR	Mucosa (grade 2)
4	48	15	CR	2.539	2.539	Alive	Mucosa (grade 2)
5	48	15	CR	971	971	Alive	Skin (grade 1), Mucosa (grade 3)
6	48	15	CR	951	951	Alive	Mucosa (grade 2)
7	48	15	CR	522	522	Alive	Skin (grade 1), Mucosa (grade 3)
8	48	15	PR	228	515	Alive	Skin (grade 1), Mucosa (grade 2)
9	48	15	CR	433	433	Alive	Skin (grade 1), Mucosa (grade 2)
10	32	10	CR	333	333	Alive	Skin (grade 1), Mucosa (grade 3)

Abbreviations: CR, complete response; Gy, grey; NTR, non‐tumour related; PR, partial response.

The tumour of the dog from Case 10 extended from the alveolus of the right mandibular canine (404), previously extracted (2 months before start of radiation therapy) by the referring veterinarian, through the mandibular canal up to M1 (409), leaving an open mandibular canal and mucosal defect at 404 following radiation therapy and complete disappearance of the initial tumour mass. Surgery was performed 132 days after completion of DRT to close the defect in order to prevent food accumulation and subsequent inflammation, osteomyelitis and necrosis of the mandible.

### 3.5 Response and survival assessment

The median follow‐up period was 961 days (range: 333‐3.498 days). Based on RECIST‐criteria treatment responses were classified as nine (9/10) complete responses and one (1/10) partial response. All dogs demonstrated substantial tumour shrinkage during radiation therapy and experienced at least a partial (4/10) or even complete (6/10) response before the end of radiation treatment.

**FIGURE 3:**
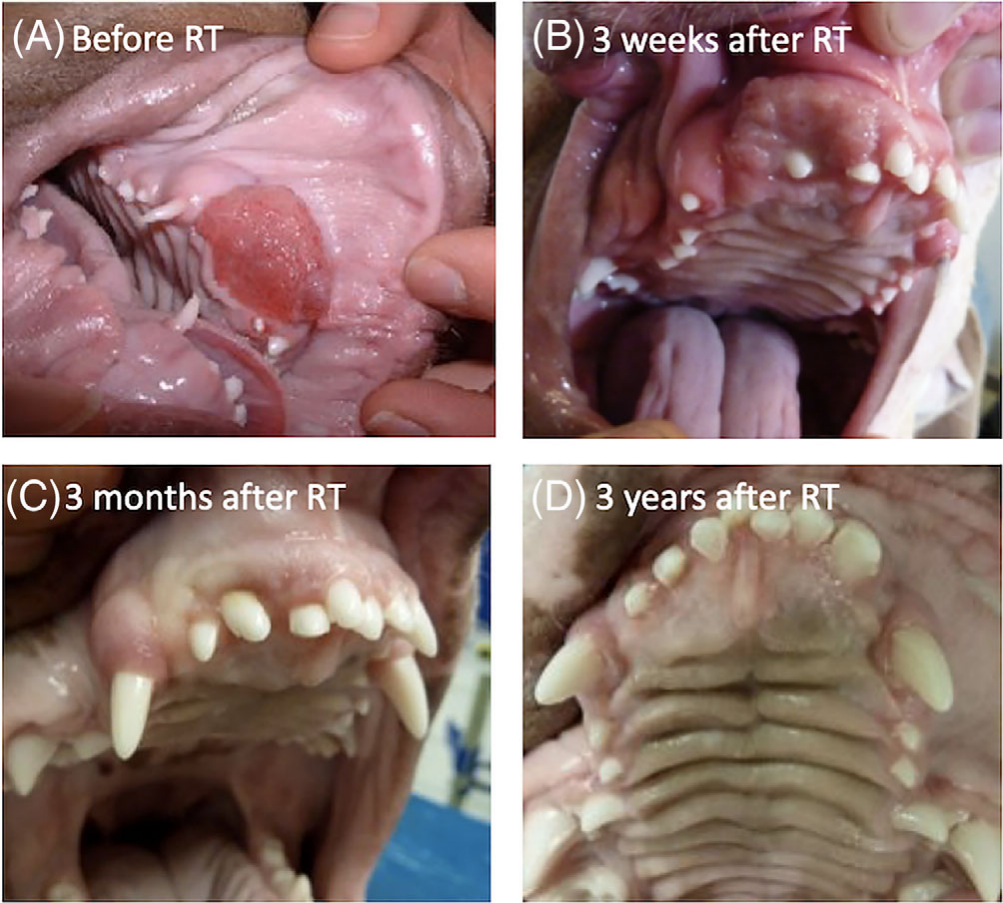
Photographs of Case 1. Note the exophytic neoplasm located in the right rostral maxilla A, prior to treatment; B, 3 weeks after radiation therapy; C, 3 months after radiation therapy; D, 3 years after radiation therapy [Colour figure can be viewed at wileyonlinelibrary.com]

**FIGURE 4:**
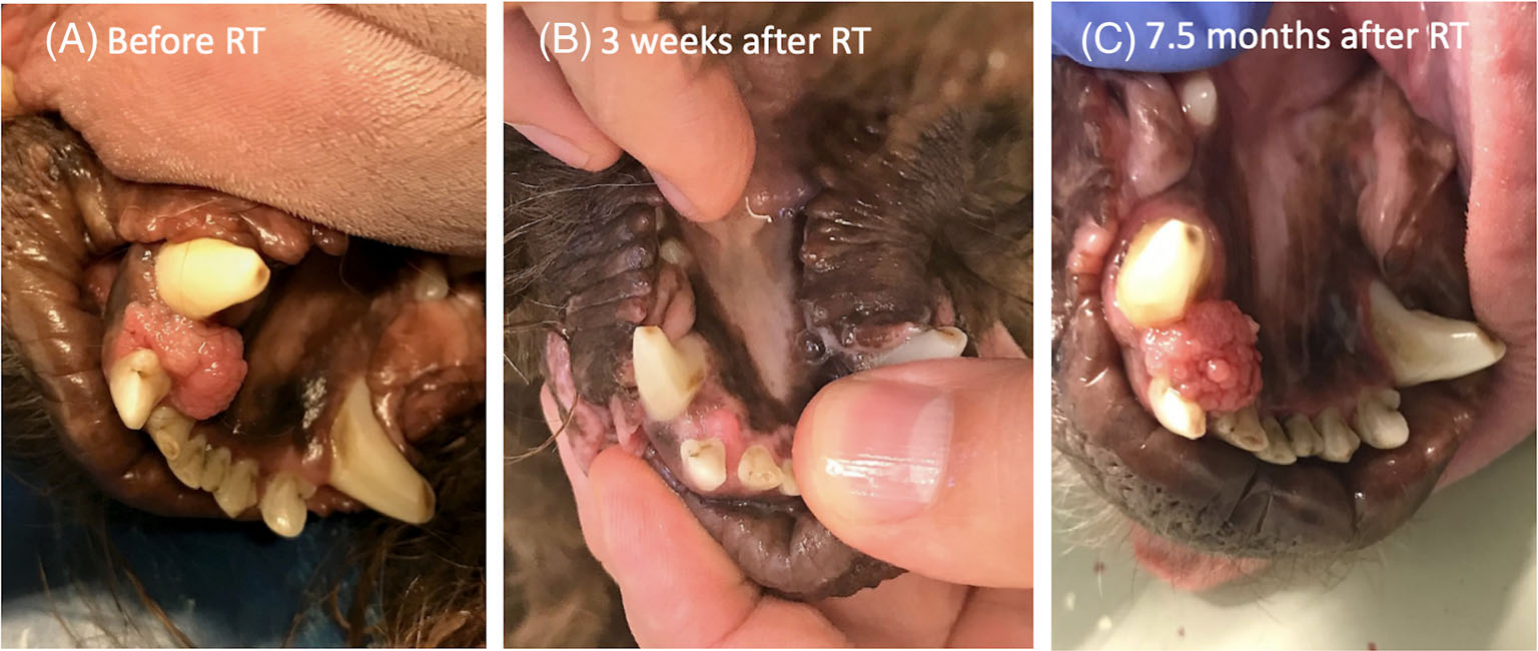
Photographs of Case 8. Note the exophytic neoplasm located in the right rostral mandible near the canine tooth A, prior to treatment; B, partial response 21 days after radiation therapy; C, progression of disease 228 days after radiation therapy [Colour figure can be viewed at wileyonlinelibrary.com]

Nine of the 10 dogs had no visible tumour mass at the 3‐week recheck (Figure 3) except for Case 8. This dog showed a near‐complete response at the 3‐week recheck and developed disease progression at 228 days after the start of radiation therapy (Figure 4). For confirmation a CT‐scan of the head and thorax were repeated, and a punch biopsy was taken for histopathology. On CT‐scan a small non‐cavitating soft tissue mass and no indication of metastatic disease was noted. Histopathology confirmed the recurrence of COPSCC. Following the punch biopsy, the owner reported the absence of macroscopic disease that persisted until the time of analysis at 287 days (515 days following the start of radiation therapy). Two dogs (Case 2 and Case 3) died from non‐tumour, non‐therapy related causes, 5 and 2 years following definitive radiation therapy respectively. Case 2 developed neurological signs (altered mental status) and died during anaesthesia for further diagnostic work‐up, whilst Case 3 was euthanized because of aggressive behaviour. These abnormalities were not considered to be radiation related because the brain was not included in the radiation field. Both dogs showed no evidence of oral PSCC recurrence at the time of death. The remaining 7 dogs were free of disease and alive at time of analysis.

Mean progression‐free survival time was 1.203 days (range: 228‐3.498), the mean overall survival time was 1.231 days (range: 333‐3.498 days) and medians were not reached at the time of analysis.

### 3.6 Toxicity

DRT was generally well tolerated. All 10 dogs experienced self‐limiting radiation‐induced oral mucositis and/or dermatitis, but no late toxicity was reported (Table 2). The acute effects were either observed during the treatment period or scored at the 3‐week recheck and consisted of grade 1 (n *= 5*) cutaneous and grade 2 (n *= 6*) or grade 3 (n *= 4*) oral mucositis. Acute effects were typically noted during the second or third week of radiation therapy. All dogs were temporarily treated with (non‐steroidal) anti‐inflammatory drugs, additional pain medication (tramadol, gabapentin) and/or antibiotics as needed. All acute effects were self‐limiting. Late effects were retrospectively evaluated in all dogs and were not reported.

## 4 DISCUSSION

This retrospective study describes the successful outcome and mild to moderate acute toxicity following the use of definitive‐intent radiation therapy as a primary treatment for macroscopic COPSCC. Dogs with oral PSCC oftentimes show a rapid and near‐complete response during radiation therapy and tumour‐inhibitory effects of the treatment lasted lifelong for the vast majority of dogs. Side‐effects were similar to those observed following radiation for other oral neoplasms and typically mild to moderate and self‐limiting. Despite the relative long follow‐up of these dogs, no late toxicity—including formation of radiation‐induced secondary malignancies—was noted. The current findings demonstrate that COPSCC is a radiosensitive tumour and suggest that this radiosensitivity is intrinsic as it appears to be independent of tumour size.

Based on RECIST criteria nine dogs (9/10) achieved a complete response and one dog (1/10) achieved a good partial response in the current study. In a previous case series, adjunct radiation therapy following surgical excision appeared effective for local tumour control.^6^ However, the current study cannot be compared with this previous study. In the current study, radiation was used as a monotherapy and tumour regression was observed shortly after the start of radiotherapy in the majority of dogs. The one dog that had only a partial response followed by tumour progression achieved permanent tumour control following punch biopsy. The reason for the initial incomplete response and tumour control after punch biopsy remains unclear. Both the initial and the recurrence histological biopsies were evaluated by two board‐certified veterinary pathologists who both agreed on the diagnosis of COPSCC. The authors have no explanation for the incomplete response to radiation for this specific tumour, but a possible explanation for the complete response following the punch biopsy might be the onset of a (late) immune response to the tumour, which might have been triggered by the previous radiation treatment.^13^, ^14^

It has been reported that differentiation between COPSCC, conventional SCC and acanthomatous ameloblastoma can be challenging.^3^, ^4^ The diagnosis of COPSCC in these patients was based on the combination of tumour appearance (exophytic papillary growth pattern), diagnostic imaging and routine histological examination. If there was any doubt regarding the histological diagnosis, biopsies were reviewed by another board‐certified veterinary pathologists. Although additional immunohistochemistry may have helped with conformation of the diagnosis, it is still no guarantee for an unequivocal diagnosis.^7^

Two distinct clinical presentations of COPSCC have been described: a cavitating pattern and a non‐cavitating pattern.^4^ Both forms have a distinct appearance in the microscopic histology, as well as their macroscopic imaging findings suggesting distinct biological behaviours of COPSCC.^4^ The current study failed to demonstrate a difference in response to radiation treatment or prognosis between these two patterns.

Eight of the ten dogs were still alive at the end of follow‐up, and therefore the median survival time was not reached. However, progression‐free and overall survival times are comparable to those reported in previous studies on COPSCC treated with aggressive surgical excision.^5^, ^8^ The results of the current study demonstrate that extended disease‐free periods are common following radiation therapy and suggest that radiotherapy is curative especially in the patients with a complete response.

Currently, the preferred primary mode of therapy for COPSCC is surgical excision.^5^, ^6^, ^8^ Most of the COPSCC affect the rostral oral cavity and can be effectively treated with wide surgical resection (1‐2 cm lateral margin) including removal of the underlying bone. Definitive‐intent radiation therapy can be an alternative treatment option in dogs with large and/or invasive COPSCC that require aggressive surgical treatment. Some of the patients in this study had a relatively small tumour and could have been treated with a curative surgical resection with minimal cosmetic or functional interference. Surgical treatment was discussed with all owners as the preferred method of treatment and only when owners declined surgical treatment definitive‐intent radiation therapy was offered as an alternative treatment option.

Although radiation therapy requires a protracted course of treatment (over 3 weeks) with multiple anaesthetic episodes and subsequent toxicity, this treatment option was preferred by all owners of the dogs included in this study.

Radiation‐induced acute toxicity was frequently noted in this study. However, signs were mild to moderate and self‐limiting, and furthermore no late toxicity was noted. Acute effects of the skin and oral mucosa are common in dogs following treatment with DRT for oral tumours.^15^, ^16^ Although acute side effects temporarily impair the quality of life, adequate analgesia proved sufficient to manage the acute side effects encountered in this study. Radiation‐induced tumour development is a potential late radiation‐induced toxicity and given its generally aggressive behaviour one of serious concern.^17^ The risk for radiation‐induced cancer is relatively low but may develop for many years following completion of the radiation therapy. In a series of 57 dogs irradiated for oral acanthomatous epulis, 2 (3.5%) dogs developed a second tumour (sarcoma, osteosarcoma) in the radiation treatment field between 5.2 and 8.7 years after the end of radiation therapy.^18^ Although the risk is relatively low, it is of particular concern in younger dogs that undergo radiation therapy for a radiosensitive tumour with the potential for very long‐term survival. Since, COPSCC appears to be a radiosensitive tumour that frequently occurs in relatively young dogs there is a potential risk for radiation induced carcinogenesis. Although no radiation‐induced cancers were observed during this study, it has to be realized that this is potentially because of the low number of dogs included in this study and the median follow‐up period being 961 days.

In this study, the intended radiation protocol was a regularly fractionated protocol consisting of 15 daily fractions with a fractional dose of 3.2 Gy (total dose of 48 Gy) and this resulted in an overall excellent response. External electron beam radiation therapy was used to spare deeper healthy tissue. Regular canine oral SCC and acanthomatous ameloblastoma are also treated with multifractionated protocols and it is recommended to use a total dose of 40 to 54 Gy administered over 10 to 20 fractions.^15^, ^19^ For COPSCC an adjunct radiation therapy protocol with a total dose of 40 Gy delivered in 10 fractions (3 fractions per week) has also been described.^6^

However, a lower fractional dose is preferred because of a reduced risk for late toxicity. The apparently high radiosensitivity of COPSCC observed in the current study suggests that less intensive treatments might be feasible. More research is necessary to determine the optimal radiation protocol for COPSCC.

This study has several limitations, most notably the small sample size and its retrospective design. Ideally, both tumour response and acute toxicity following completion of radiation therapy would have been evaluated on a weekly basis by the same clinician. Although this was not carried out, owners were instructed to contact the Oncology service in case of worrisome adverse events, such as, discomfort, pain or infection. This information was included in the toxicity scoring. However, it may have resulted in underestimation of both frequency and degree of acute radiation toxicity. Since, late effects were retrospectively evaluated by review of the medical records and telephone interviews this might also have led to the situation that mild late toxicity, such as, changes in mucosal pigmentation, alopecia and leukotrichia were under reported.

In conclusion, results of the present study indicate that multifractionated radiation therapy is highly effective in treating macroscopic COPSCC. Radiation treatment is well tolerated, and durable complete tumour responses are observed in the majority of dogs. Although further studies are needed to confirm the observed excellent treatment response, evaluate possible prognostic factors, determine the optimal treatment protocol and allow for longer follow‐up time in order to objectively assess the risk of tumour recurrence or radiation‐induced tumour formation, our findings suggest that multifractionated radiation therapy is a viable monotherapy for COPSCC and a realistic alternative to surgery especially in those cases where aggressive surgery is not possible or not acceptable for the owner.

## CONFLICT OF INTEREST

The authors declare no potential conflict of interest.

## Data Availability

The data that support the findings of this study are available from the corresponding author, [F.E.M.M. van der Steen], upon reasonable request.
